# Electronic cigarette vapour moderately stimulates pro-inflammatory signalling pathways and interleukin-6 production by human monocyte-derived dendritic cells

**DOI:** 10.1007/s00204-020-02757-8

**Published:** 2020-05-05

**Authors:** I-Ling Chen, Ian Todd, Patrick J. Tighe, Lucy C. Fairclough

**Affiliations:** School of Life Sciences, University of Nottingham, Life Sciences Building, University Park, Nottingham, NG7 2RD UK

**Keywords:** Dendritic cell, Electronic cigarette, Interleukin-6, Signalling molecule, Protein array

## Abstract

Dendritic cells (DCs) are professional antigen presenting cells that play a critical role in bridging innate and adaptive immunity. Numerous studies have shown that tobacco constituents present in conventional cigarettes affect the phenotype and function of DCs; however, no studies have examined the effects of vapour from E-cigarettes on human DCs. Here, the effects of E-cigarette vapour extract (ECVE) on the phenotype and function of DCs were investigated by creating an in vitro cell culture model using human monocyte-derived DCs (MoDCs). Immature DCs were generated from peripheral blood monocytes and mature DCs were then produced by treatment with LPS or Poly I:C for 24 h. For LPS-matured DCs, 3% ECVE treatment slightly suppressed HLA-DR and CD86 expression, whereas 1% ECVE treatment enhanced IL-6 production. The overall expression of 29 signalling molecules and other cytoplasmic proteins (mainly associated with DC activation) was significantly upregulated in immature DCs by 1% ECVE, and in LPS-treated DCs by 3% ECVE. In particular, the condition that induced IL-6 production also upregulated MAPK pathway activation. These findings indicate that E-cigarette vapour moderately affects human DCs, but the effects are less pronounced than those reported for tobacco smoke.

**Electronic supplementary material:**

The online version of this article (10.1007/s00204-020-02757-8) contains supplementary material, which is available to authorized users.

## Introduction

Electronic cigarettes (E-cigarettes) are handheld electronic devices that generate vapours or aerosols by heating E-liquid. The main components of E-liquids are humectants [i.e. propylene glycol (PG), vegetable glycerol (VG)], nicotine and flavourings. E-liquid is stored in the cartridge and then supplied to the atomizer which contains a small coil of electrically resistant wire that heats up when the battery is switched on. E-cigarettes have gained rapidly in popularity around the world in recent years; sales of E-cigarettes are predicted to reach $44 billion by 2023 (Research and Markets 2018). Since E-cigarettes were invented in 2003, their usage has increased dramatically: in 2018, overall usage by adults in the USA was 7.6%, but this increases to 36.5% in ex-cigarette smokers (Dai and Leventhal 2019); amongst adolescents (16–19 years) in the USA, UK and Canada, between 32 and 37% used e-cigarettes in 2018 (Hammond et al. 2019). In some countries, E-cigarettes are promoted as a tool for smoking cessation (Beard et al. 2016; Etter 2010; Polosa et al. 2011, 2014) and as a safe alternative to conventional cigarettes due to lower levels of carcinogens and toxicants in vapours (Goniewicz et al. 2014). However, the use of E-cigarettes remains controversial (Bush et al. 2019). A rapid rise in E-cigarette use has occurred not only amongst current smokers, but also non-smokers who may, therefore, develop a smoking habit and/or nicotine addiction (Lam et al. 2014). Although it is still too soon to know the effects of chronic E-cigarette use over a period of decades, there have been several reports since 2014 of acute lung injury, pneumonitis and/or pneumonia associated with E-cigarette use (Arter et al. 2019; Khan et al. 2018; Layden et al. 2019; Moore 2015; Nair et al. 2019; Sommerfeld et al. 2018; Thota and Latham 2014).

E-cigarettes have been found to have effects on a variety of cell types in humans, including epithelial and endothelial cells, fibroblasts, macrophages and neutrophils (Chen et al. 2019). However, the effects of E-cigarette vapours (ECV) on human myeloid DCs (mDCs) have not been studied previously. In contrast, numerous studies have shown that the phenotype and functions of mDCs are affected by tobacco smoke, with suppression of T cell-stimulating, immunogenic properties of mDCs by CS constituents (Stampfli and Anderson 2009). Evidence for this derives from human and murine in vivo and in vitro studies. In a mouse smoking model, DCs show low expression of co-stimulatory molecules and reduced stimulation of T cells (Robbins et al. 2004, 2008). Similarly, in humans, smoking impairs the maturation of mDCs in the lungs, evidenced by reduced expression of CD40, CD83 and CCR7 (Arellano-Orden et al. 2016; Liao et al. 2015). Treatment of mouse bone marrow-derived DCs (bmDCs) with cigarette smoke extract (CSE) differentially affects T cell stimulation, depending on T cell phenotype (CD4^+^ or CD8^+^) (Mortaz et al. 2009) and length of treatment (chronic exposure to CSE suppresses DC maturation) (Givi et al. 2015). The maturation of human monocyte-derived DCs (moDCs), and their ability to stimulate Th1 and Th17 cells, is also suppressed by CSE (Kroening et al. 2008; Le Rouzic et al. 2016; Vassallo et al. 2005). We have shown that CSE treatment of human moDCs reduced their expression of pro-inflammatory signalling molecules (Alkhattabi et al. 2018). Although numerous chemicals within CS affect immune cells (Stampfli and Anderson 2009), nicotine is the main addictive component and has well-documented suppressive effects on IL-12 production and Th1 stimulation by human moDCs and mouse bmDCs, promoting instead Th2 responses (Guinet et al. 2004; Nouri-Shirazi and Guinet 2003, 2006; Nouri-Shirazi et al. 2007). Immunosuppressive effects of nicotine are exerted particularly via the α7 nicotinic acetylcholine receptor (Zdanowski et al. 2015). It should also be noted that some studies indicate the activation of DCs by CS (Bratke et al. 2008; Vassallo et al. 2008, 2010).

Although some studies have reported that CS and ECV have similar effects on various cell types (Chen et al. 2019), others have found that the effects of CS are much more pronounced and deleterious than those of ECV (Husari et al. 2016; Iskandar et al. 2019; Taylor et al. 2016). Therefore, in view of the critical role of DCs in immunity and the marked effects of CS on DCs, in the current study, the effects of ECV on the phenotype and function of DCs was investigated by creating an in vitro cell culture model using human moDCs. Human moDCs were treated with E-cigarette vapour extract (ECVE) and the effects of ECVE on surface marker expression, cytokine secretion and signalling pathways was examined.

## Materials and methods

### Generation of monocyte-derived DCs

Fresh venous blood was taken into heparinised Vacutainer tubes from healthy, never-smoking volunteers with ethical approval (FMHS REC ref: 121-1706) and informed consent. Peripheral blood mononuclear cells (PBMCs) were separated by density gradient centrifugation using Histopaque-1077 (Sigma-Aldrich, Gillingham, UK). The cells were washed and resuspended in RPMI complete medium, that is RPMI-1640 medium supplemented with 100 μg/ml Penicillin–Streptomycin (Sigma-Aldrich, Gillingham, UK), 2 mM l-Glutamine (Sigma-Aldrich, Gillingham, UK), 10 mM HEPES (Sigma-Aldrich, Gillingham, UK) and 10% fetal bovine serum (FBS, Sigma-Aldrich, Gillingham, UK).

CD14 + monocytes were isolated from PBMCs using CD14 + microbeads (Miltenyi Biotec, Bisley, UK) by positive selection according to the manufacturer’s instructions. One ml of CD14 + cell suspension (5 × 10^5^ cells) was added to each well in a 24-well plate (Costar, High Wycombe, UK) in the presence of 50 ng/ml GM-CSF (Peprotech, London, UK) and 400 IU IL-4 (Peprotech, London, UK) and incubated at 37℃, 5% CO_2_ for 5 days. Fresh medium and cytokines were added on day 3. To generate mature DCs, immature DCs on day 5 were stimulated with 100 ng/ml LPS or 30 μg/ml Poly I:C (Sigma-Aldrich, Gillingham, UK) for 24 h.

### ECVE preparation

E-cigarette devices and a DIY E-liquid Mixing Kit were purchased from VAPEMATE (Brentwood, UK). The E-liquid contained 50/50 PG/VG and 12 mg/ml nicotine. The smoking apparatus consisted of a three-way stopcock attached to a 50 ml syringe, the E-cigarette and a bottle containing 20 ml RPMI complete medium. The vapour was drawn into the syringe and then bubbled into the 20 ml RPMI complete medium at a flow rate of 50 mL/min. It took approximately 25 cycles to consume 200 mg of E-liquid. The medium was then filter-sterilized with a 0.45 μm pore-size filter. The EC cartridge was weighed before and during the process to confirm that 200 mg of liquid was consumed. The resulting extract was considered as 100% ECVE. Further dilution was needed to generate 1% and 3% ECVE, which were used to treat DCs. The choice of final ECVE concentrations was based on nicotine content as follows: the E-liquid contained 12 mg of nicotine per gram of liquid. Therefore, 200 mg of E-liquid contained 2.4 mg nicotine. This resulted in 1% ECVE containing approximately 12 μg/ml nicotine and 3% ECVE containing 36 μg/ml nicotine. This is similar to the nicotine concentrations in 0.5–3% cigarette smoke extract (CSE) used in previous studies (Alkhattabi et al. 2018).

### Treatment of MoDCs with ECVE

On Day 5, immature MoDCs were cultured with or without 100 ng/ml lipopolysaccharide (LPS) or 30 µg/ml polyinosinic polycytidylic acid (Poly I:C) (Sigma-Aldrich, Gillingham, UK), in the absence or presence of 1% or 3% ECVE, at 37 °C, 5% CO_2_ for 24 h. The cells treated with LPS or Poly I:C for 24 h (with or without simultaneous treatment with ECVE) are referred to as ‘LPS-matured DCs’ or ‘Poly I:C-matured DCs’, respectively.

### Extracellular staining for surface marker detection

DCs were placed in 1 ml PBA buffer (PBS, 1% BSA and 0.1% sodium azide) and centrifuged at 300 g for 5 min. The supernatant was discarded and the cell pellets were resuspended. Samples were incubated 30 min in the dark at room temperature with fluorochrome-labelled antibodies (as indicated in Table 1); corresponding isotype control antibodies were also applied. After incubation, 2 ml of PBA was added to remove excess and unbound antibodies, followed by centrifugation at 300 g for 5 min. Finally, the cells were fixed with 0.5% formaldehyde and the samples were kept at 4 °C until flow cytometric analysis. Flow cytometry was carried out using an FC500 Flow Cytometer (Beckman Coulter) and data were analysed using Weasel Software v3.0.2.

**Table 1:** Antibodies used for flow cytometric analysis and their conjugation and manufacturers

Antibody	Clone	Isotype	Fluorochrome	Company
CD80	IM1853U	IgG1	FITC	Beckman Coulter
DC-SIGN	A07407	IgG1	PE	Beckman Coulter
CD86	REA968	IgG1	FITC	Miltenyi Biotec
CD83	REA714	IgG1	PE	Miltenyi Biotec
HLA-DR	REA805	IgG1	APC	Miltenyi Biotec
CD40	REA733	IgG1	PE-Vio 770	Miltenyi Biotec
CD36	REA760	IgG1	FITC	Miltenyi Biotec
CXCR-4	REA649	IgG1	APC	Miltenyi Biotec
CD74	REA1103	IgG1	PE-Vio 770	Miltenyi Biotec
PD-L1	MIH1	IgG1	APC	BD Biosciences

### Enzyme-linked immunosorbent assay (ELISA) for cytokine detection

Cell-free culture supernatants were collected and stored at − 20 °C before analysis. The levels of cytokines IL-6, IL-8, IL-10, IL-12p70, and TNF-α were measured by sandwich ELISA using Duo Set ELISA kits (R&D Systems) according to the manufacturer’s instructions.

### Reverse-phase protein microarray (RPPA)

RPPA was performed as described previously (Alkhattabi et al. 2018; Negm et al. 2014). We had previously confirmed that the detection of signalling molecules by RPPA gave results consistent with those obtained by western blotting (Negm et al. 2014). DCs were harvested and centrifuged for 5 min at 300*g*. The pellets were resuspended in RIPA buffer (Thermo Scientific, Loughborough, UK), supplemented with phosphate/protease inhibitor (Thermo Scientific, Loughborough, UK) and benzonase nuclease ultrapure (Sigma-Aldrich, Gillingham, UK). Samples were then incubated on ice for 30 min with frequent shaking followed by centrifugation at 10,000*g* at 4 °C for 10 min. The supernatants were collected and stored at − 80 °C until assayed.

Before conducting RPPA, a BCA protein assay was performed using a Micro BCA™ Protein Assay Kit (Thermo Scientific, Loughborough, UK) to confirm the protein concentration of lysates. Lysates were diluted to 500 μg/ml with 4 × SDS printing buffer containing 0.4 M DTT and heated at 95 °C for 5 min. Subsequently, lysates were transferred to 394-well plates and were robotically spotted onto nitrocellulose-coated glass slides by microarrayer (MicroGrid II, Digilab) and dried in the air. Printed slides were stored at − 20 °C until they were processed.

Slides were incubated in Super G blocking buffer (Grace Bio-Labs) for 1 h. After washing with 0.5% Tween-20 in PBS 3 times for 5 min each time, slides were incubated with primary antibodies (Table 2) diluted in blocking buffer and incubated for 2 h at room temperature. Mouse anti-β-actin (Sigma Aldrich, Gillingham, UK), diluted 1:1000 in blocking buffer, was used to monitor this ‘housekeeping’ protein to control for differences in protein loading. After washing 3 times with 0.5% Tween-20 in PBS, the slides were incubated with biotinylated secondary antibodies diluted 1:20,000 in blocking buffer and incubated for 2 h at room temperature. Next, the slides were incubated with streptavidin conjugated to infrared dyes, IRDYE-800CW (1:10,000 in blocking buffer, LI-COR Biotechnology, Cambridge, UK) for 30 min at room temperature. Lastly, the slides were rinsed with distilled water, centrifuged dry and scanned with a Licor Odyssey scanner (LI-COR Biotechnology, Cambridge, UK) at 800 nm. The resultant TIFF images were processed with GenePix Pro-6 Microarray Image Analysis software (Molecular Devices). Protein signals were finally determined using background subtraction and normalization to the internal housekeeping targets.

**Table 2:** Primary antibodies used in RPPA specific for the proteins of interest in DC lysates

Antibody specificity	Species	Dilution	Antibody specificity	Species	Dilution
c-Rel (12707)	Rabbit	1:200	pSTAT3 (9145)	Rabbit	1:50
pMST1 (3681)	Rabbit	1:200	pHSP27 (2401)	Rabbit	1:50
HMGB1 (6893)	Rabbit	1:200	p44/42 MAPK (4370)	Rabbit	1:250
LSP1 (3812)	Rabbit	1:100	pAKT Threonine (9275)	Rabbit	1:50
nNOS (4231)	Rabbit	1:100	pAKT Serine (9271)	Rabbit	1:50
PIPK1C (3296)	Rabbit	1:100	pC-Jun (9261)	Rabbit	1:250
CD74 (77274)	Rabbit	1:400	pNF-κB P65 (3031)	Rabbit	1:50
IRAKM (4369)	Rabbit	1:50	pSTAT1 (9177)	Rabbit	1:250
TAB2 (3744)	Rabbit	1:250	pROS (3078)	Rabbit	1:250
IRF3 (4302)	Rabbit	1:2000	pc-Fos (5348)	Rabbit	1:500
pIRF3 (4947)	Rabbit	1:2000	Bcl-xL (2764)	Rabbit	1:250
pTAB2 (8155)	Rabbit	1:500	IRAK1 (4362)	Rabbit	1:250
NALP1 (4990)	Rabbit	1:500	IRAK2 (4367)	Rabbit	1:50
pIRF7 (5184)	Rabbit	1:500	pP38 (4511)	Rabbit	1:2000
SOD1 (4266)	Mouse	1:400			

All antibodies were purchased from Cell Signalling Technology

Changes within the expression of many signalling molecules takes place within a few minutes of cell stimulation. To determine the peak of signalling molecule expression, a time course experiment was performed. DCs were treated with LPS or Poly I:C and ECVE for the following time durations: 5, 15, 30, 60 and 120 min and expression of signalling molecules examined by RPPA. The expression of many signalling molecules peaked at 30 min, followed by a reduction of signal level in both immature and maturing DCs. Therefore, in addition to 24 h-ECVE treatment, 30-min ECVE treatment was employed to study the effects of ECVE treatment on signalling pathways in DCs.

### Statistical analysis

Data were analysed using GraphPad Prism v7.02 for Windows (GraphPad Software) and values expressed as median from independent experiments, unless otherwise stated. A test for normality was performed and either paired *t* test/Wilcoxon matched-pairs test or paired one-way ANOVA/paired Friedman test was performed, as appropriate to the data. A *p* value < 0.05 was considered statistically significant.

## Results

### The effects of ECVE on surface markers of MoDCs

**Fig. 1:**
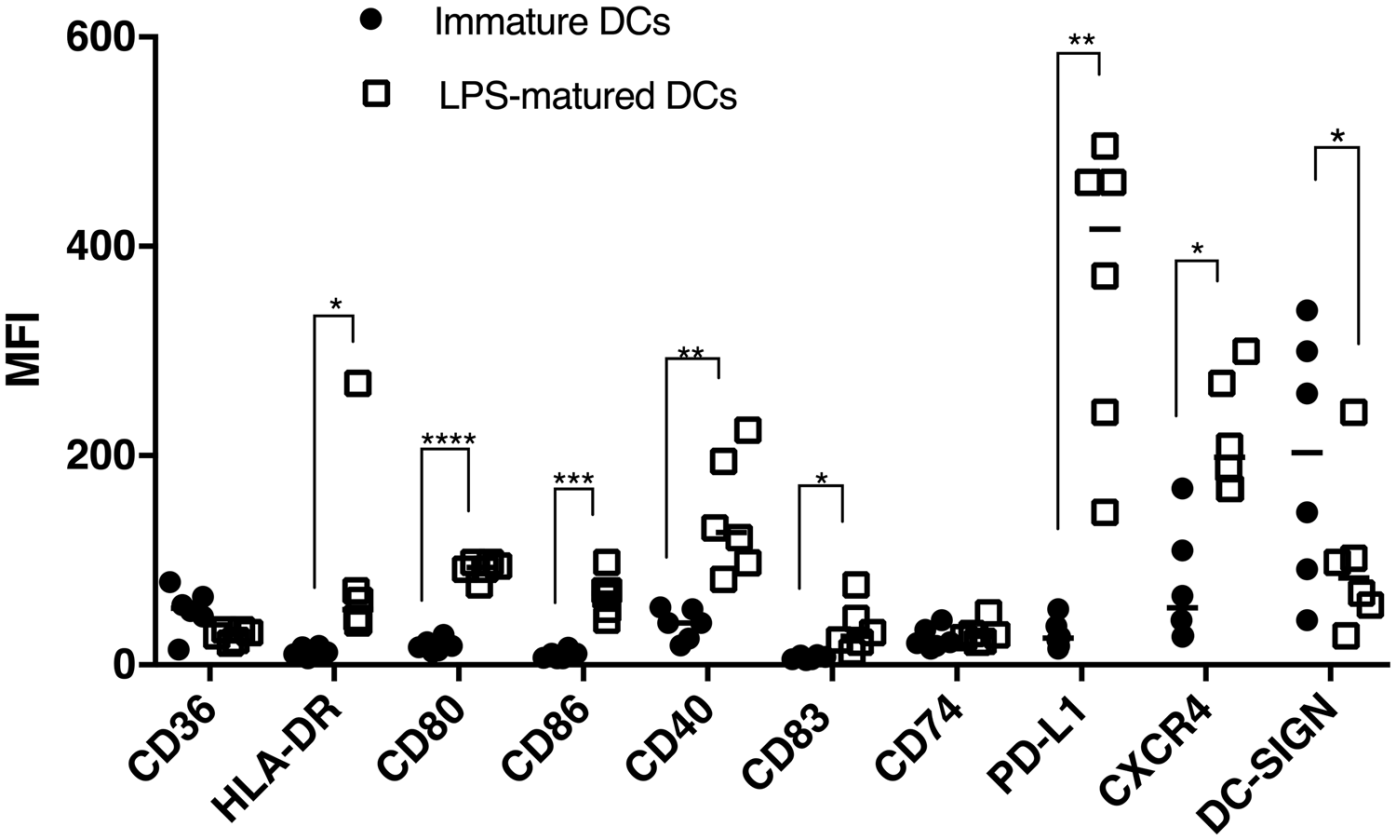
Comparison of MFI value of surface markers between untreated immature and LPS-matured DCs. Data are presented as scatter plots and each dot represents a different individual donor. The median of six independent experiments is shown. If data were normally distributed, paired *t* test was used, otherwise Wilcoxon test was used. *p < 0.05, **p < 0.01, ***p < 0.001, ****p < 0.0001

The generation of mature DCs by treatment of MoDCs with LPS was confirmed by changes in surface markers expression using flow cytometry. Stimulation of MoDCs with 100 ng/ml LPS for 24 h significantly elevated surface expression of HLA-DR, CD80, CD86, CD40, CD83, PD-L1 and CXCR4, but decreased expression of DC-SIGN (Fig. 1).

**Fig. 2:**
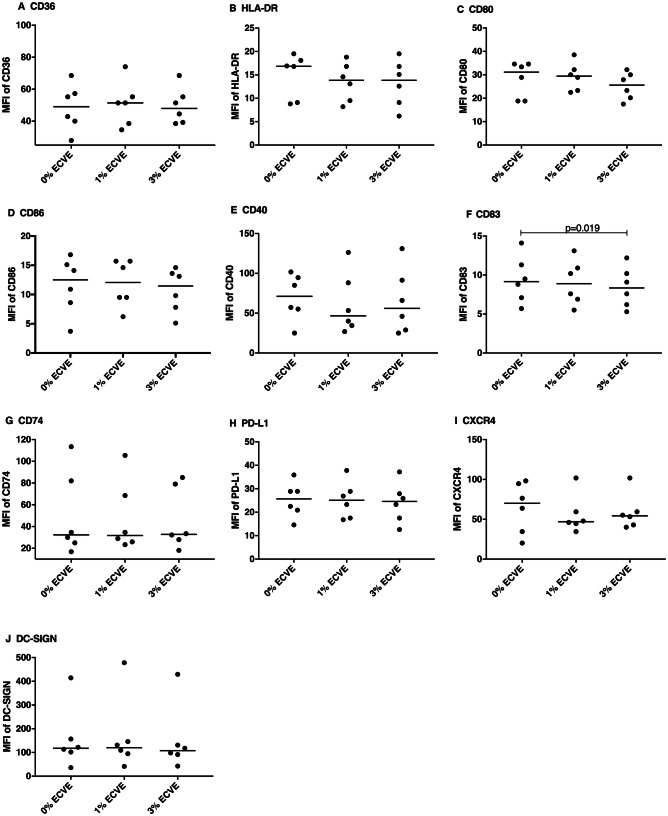
The MFI of surface markers on immature DCs after 24 h with or without ECVE treatment. Data are presented as scatter plots and each dot represents a different individual donor. The median of six individual experiments is shown. If data were normally distributed, paired one-way ANOVA was used, otherwise paired Friedman test was used

**Fig. 3:**
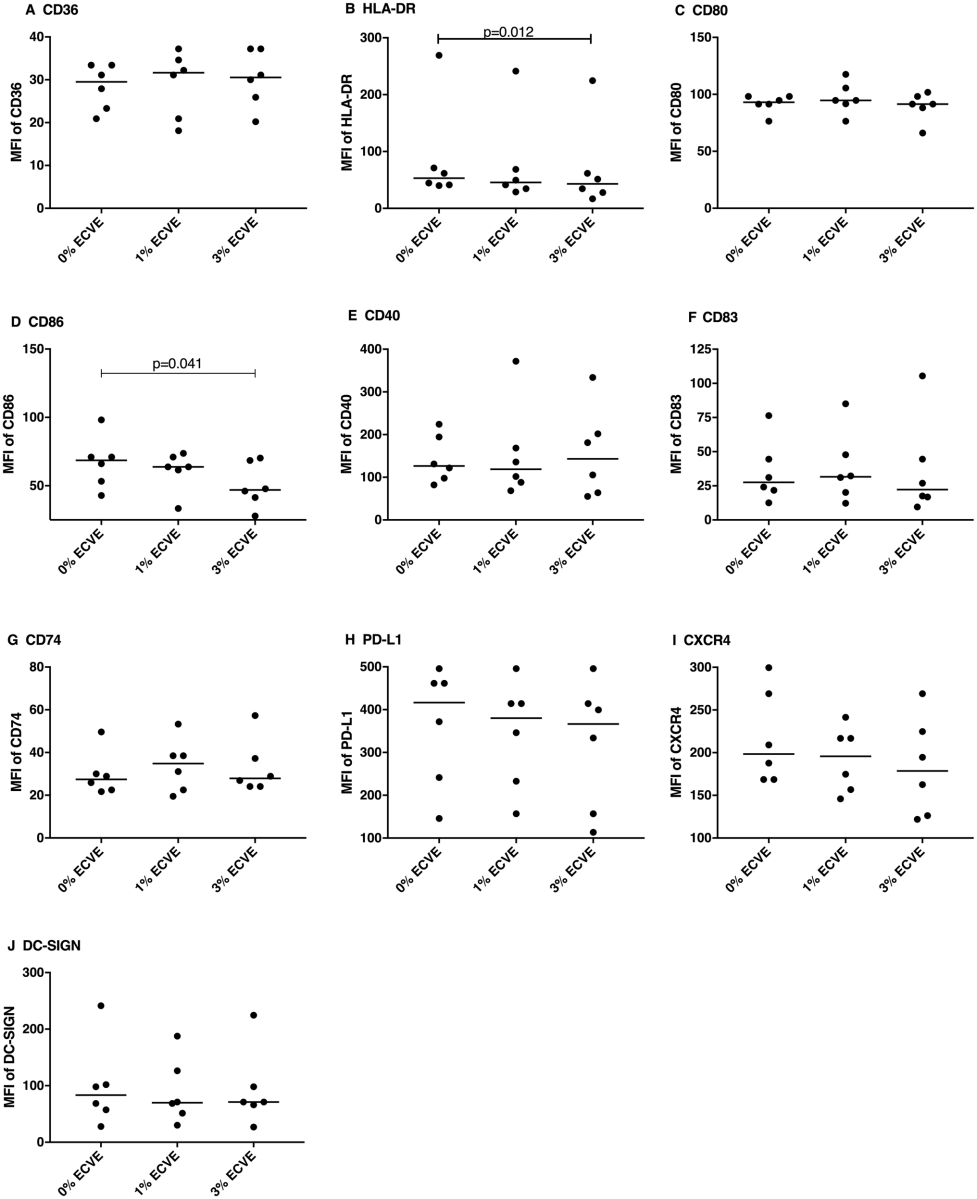
The MFI of surface markers on LPS-matured DCs after 24 h with or without ECVE treatment. Data are presented as scatter plots and each dot represents a different individual donor. The median of six individual experiments is shown. If data were normally distributed, paired one-way ANOVA was used, otherwise paired Friedman test was used

To determine the effect of ECVE on surface marker expression, immature and LPS-matured DCs were treated with or without ECVE for 24 h (Figs. 2 and 3, respectively); ECVE treatment did not significantly reduce the viability of moDCs (data not shown). The expression of CD83 on immature DCs was slightly, but significantly reduced after 3% ECVE treatment for 24 h (Fig. 2). Treatment with 3% ECVE slightly, but significantly, inhibited the expression of HLA-DR and CD86 on LPS-matured DCs (Fig. 3). No alterations were found in the expression of other surface markers in response to ECVE treatment.

Stimulation of MoDCs with 30 μg/ml Poly I:C for 24 h also significantly elevated surface expression of HLA-DR, CD80, CD86, CD40, CD83 and PD-L1 (but not CXCR4), and decreased expression of DC-SIGN (Supplementary Fig. 1S). The treatment of Poly I:C-matured DCs with 1% or 3% ECVE did not alter significantly the expression of any of the surface markers examined (Supplementary Fig. 2S).

### The effects of ECVE on cytokine production by MoDCs

**Fig. 4:**
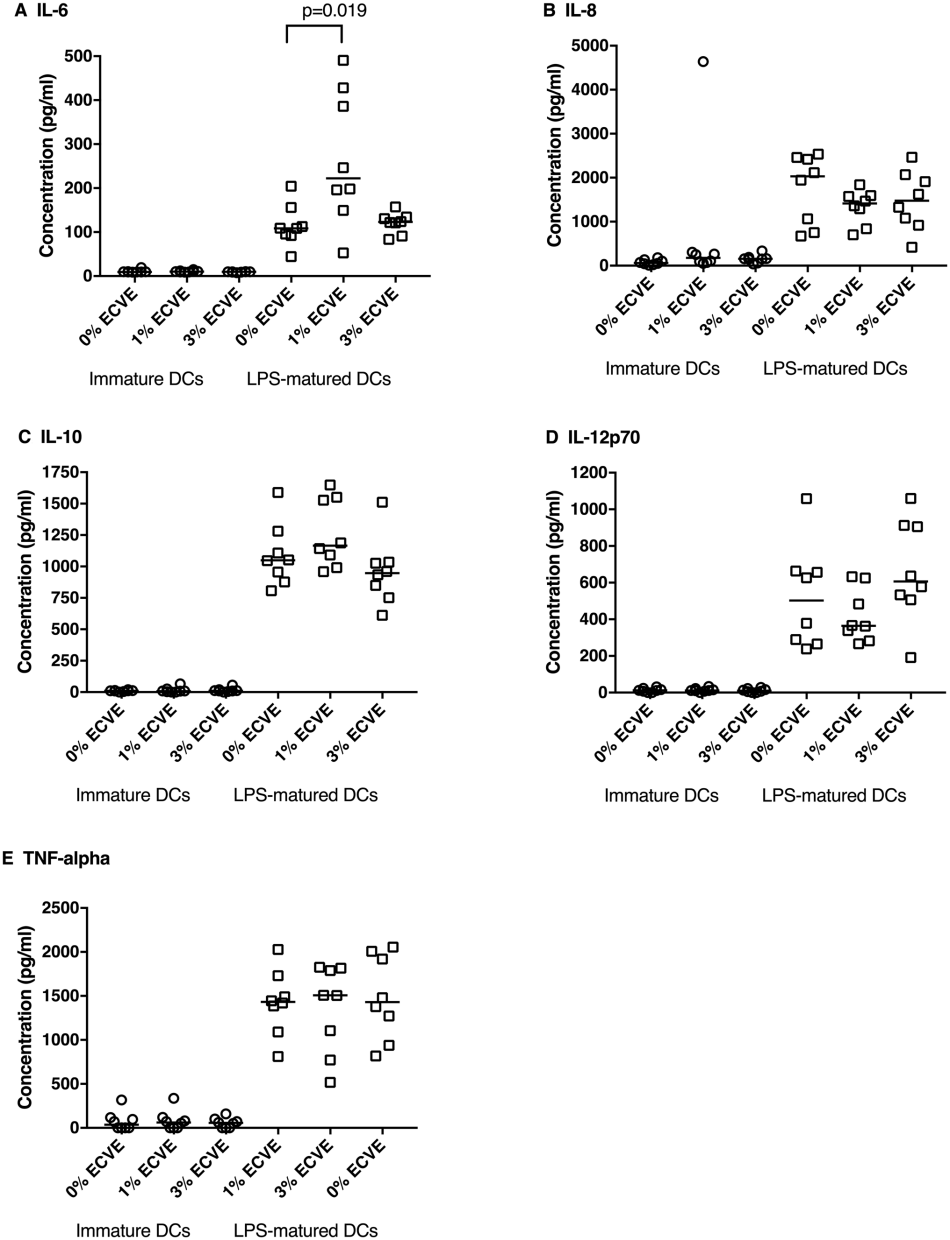
Cytokine production by immature and LPS-matured DCs after 24 h with or without ECVE treatment. Data are presented as scatter plots of eight independent experiments and their medians. If data were normally distributed, paired one-way ANOVA was used, otherwise paired Friedman test was used

The culture supernatants of immature and LPS-matured DCs, cultured in the absence or presence of 1% or 3% ECVE for 24 h, were assayed by ELISA to determine levels of IL-6, IL-8, IL-10, IL-12p40 and TNF-α. Twenty-four hour ECVE treatment did not affect the secretion of these cytokines from immature DCs. For LPS-matured DCs, 1% (but not 3%) ECVE significantly enhanced the production of IL-6 (Fig. 4). Levels of IL-8, IL-10, IL-12p70 and TNF-α were unaffected by ECVE treatment.

The treatment of Poly I:C matured DCs with 1% or 3% ECVE for 24 h did not significantly alter secreted levels of any of the cytokines assayed (IL-6, IL-8, IL-10, IL-12p70 or TNF-α) (Supplementary Fig. 3S).

### The effects of ECVE on signalling molecule expression of MoDCs

**Fig. 5:**
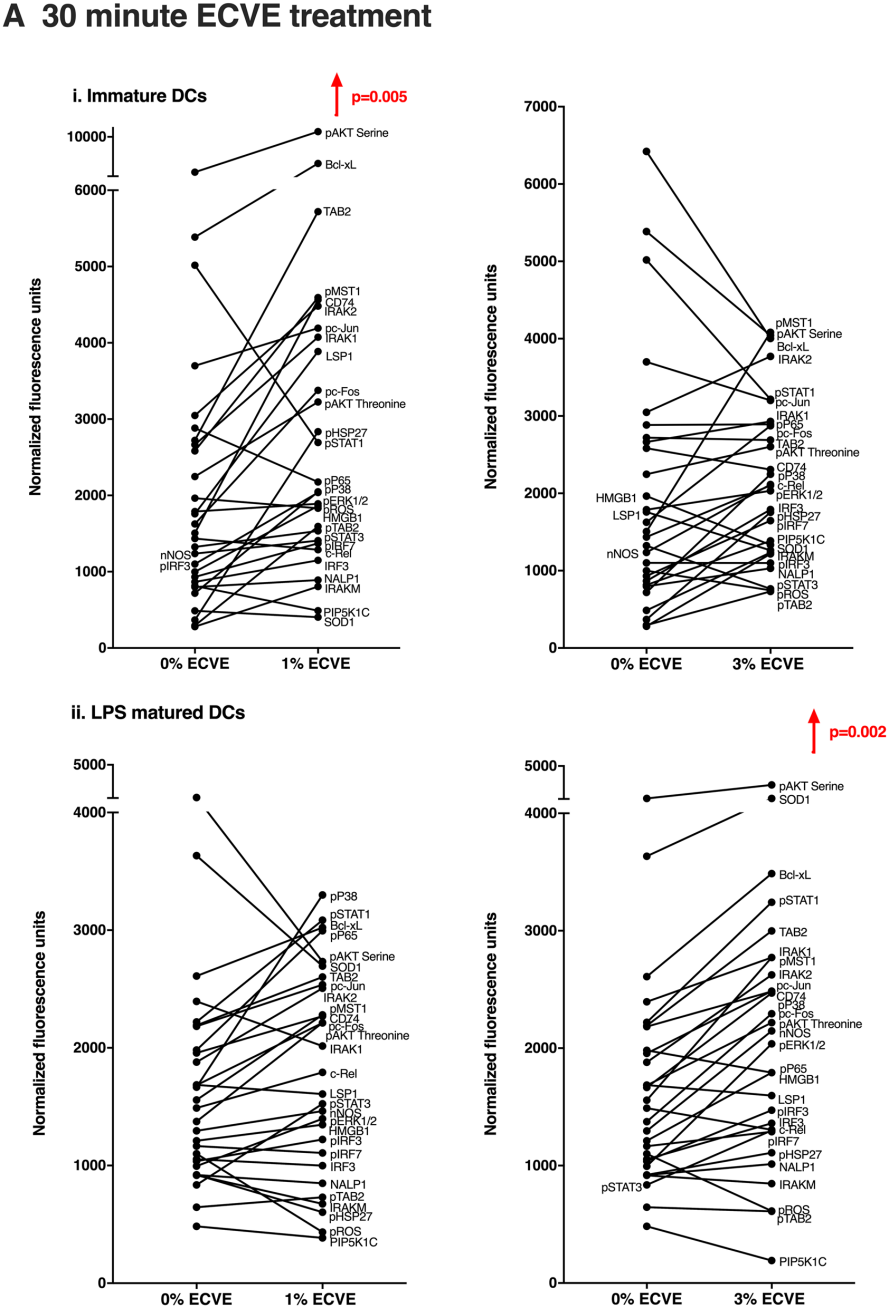

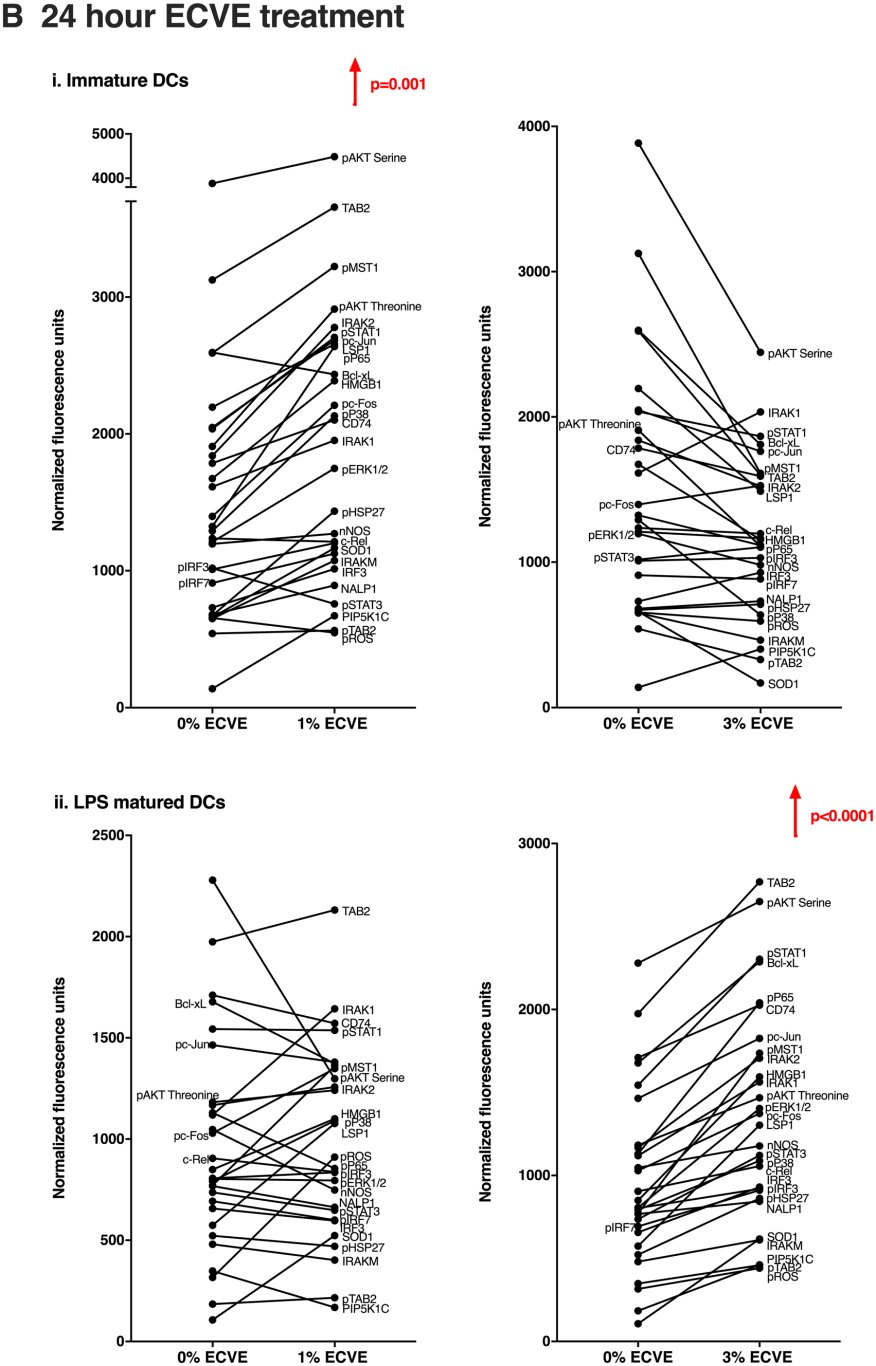
The effects of ECVE treatment on global expression of signalling molecules by DCs. Global effects on 29 signalling molecules in DCs treated with ECVE for **a** 30 min and **b** 24 h. Data are presented as scatter plots and each dot represents the median expression of each signalling molecule from up to nine independent experiments. If data were normally distributed, one-way ANOVA was used; otherwise Freidman test was used

RPPA was used to analyse the expression by DCs of 29 signalling molecules and other cytoplasmic molecules mainly associated with DC activation and immunogenicity; several of the molecules are involved in IRAK/IRF, MAPK, NF-κB, JAK/STAT and PI3K/AKT pathways, amongst others (see Table 2). Thirty-minute or 24 h ECVE treatment did not cause a statistically significant change in the expression of most individual molecules of immature, LPS-matured or Poly I:C-matured DCs (data not shown). However, it was observed that, for many molecules, ECVE treatment gave a trend towards higher molecule expression. We therefore determined the combined or ‘global’ effects of ECVE on all 29 molecules investigated by comparing the median expression (for up to 9 donors) of all molecules in the absence or presence of ECVE. The basis for this is that biological effects are likely to depend on the cumulative changes in expression of multiple molecules associated with signalling pathways, although the change for each individual molecule may be relatively subtle. 1% ECVE treatment was then seen to increase significantly the global expression of the molecules in immature DCs after 30 min (Fig. 5a) and 24 h (Fig. 5b). 3% ECVE treatment also significantly increased global expression of the molecules in LPS-maturing DCs after 30 min (Fig. 5a) and in LPS-matured DCs after 24 h (Fig. 5b).

For Poly I:C-treated DCs, 1% ECVE treatment for 30 min significantly reduced the ‘global’ signalling molecule expression (Supplementary Fig. 4S). However, treatment with 3% ECVE for 30 min, or either 1% or 3% ECVE for 24 h had no significant effect on global signalling molecule expression (Supplementary Fig. 4S).

**Fig. 6:**
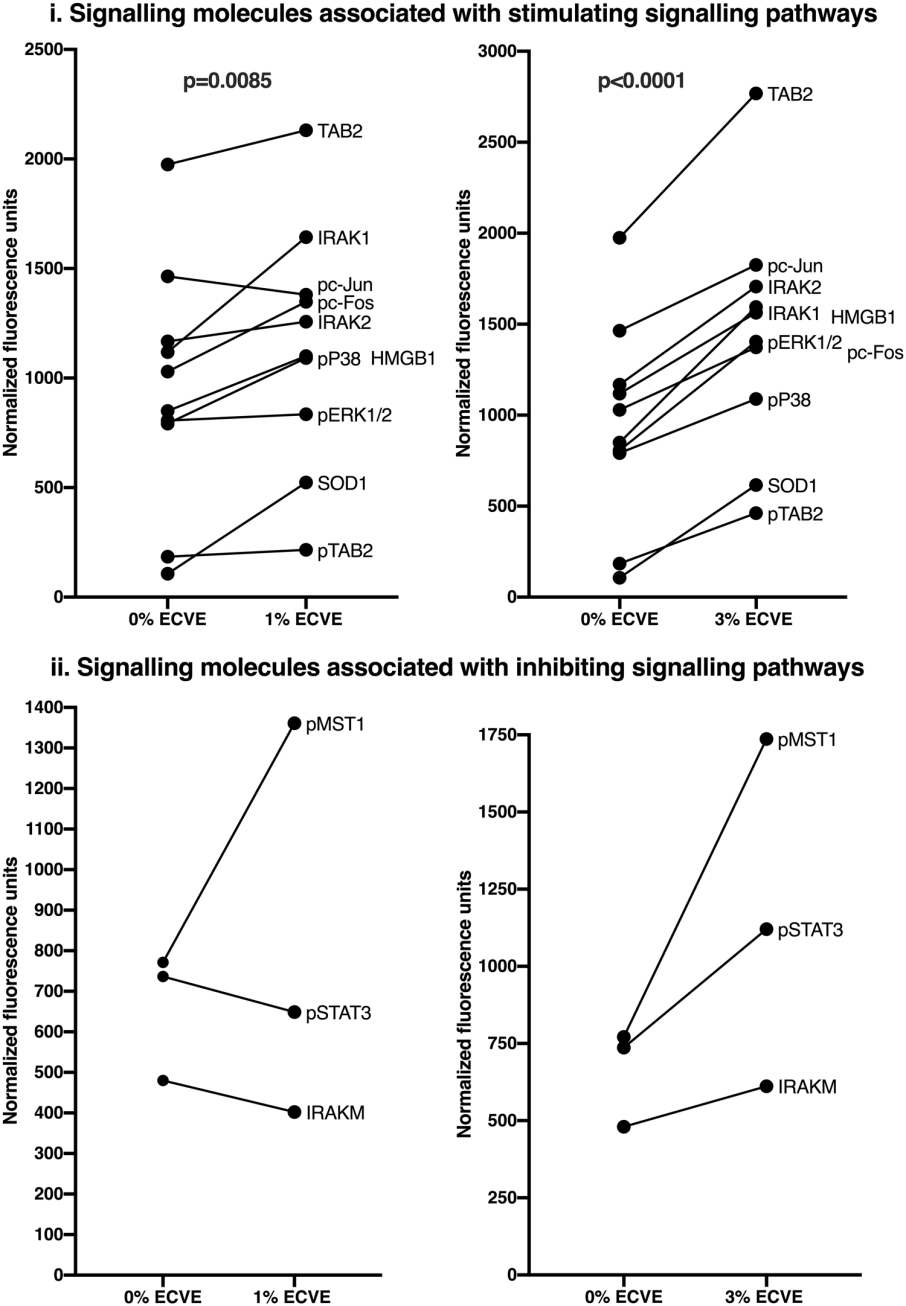
Selected RPPA data for LPS-matured DCs treated with 1% or 3% ECVE. The median values presented are taken from Fig. 5. Data are presented as scatter plots and each dot represents the median expression of each signalling molecule from up to nine independent experiments. If data were normally distributed, paired *t* test was used; otherwise Wilcoxon test was used. In (i): for 0% versus 1% ECVE, *p* = 0.0085; for 0% versus 3% ECVE, *p* < 0.0001

We observed that many of the molecules upregulated by 24 h treatment of LPS-matured DCs with 1% ECVE (i.e. the conditions in which ECVE treatment significantly enhanced IL-6 production) were those associated with the IRAK/TRAF6/TAB pathway that is stimulated when LPS binds to TLR4, and which leads to MAPK activation and IL-6 production: these molecules are IRAK1, IRAK2, TAB2, pTAB2, pP38, pERK1/2 and pC-Fos (Fig. 6i). In addition, the molecules HMGB1 and SOD1, which can further stimulate this pathway are presented (Fig. 6i). Conversely, certain molecules that were also measured can have an inhibitory effect on the pathway (i.e. IRAK-M, pSTAT-3, pMST-1) (Fig. 6ii). The median values for all of these molecules (from up to 9 donors) are plotted: this shows that both 1% ECVE (Fig. 6i, left panel) and 3% ECVE (Fig. 6i, right panel) significantly up-regulated the signalling (and other) molecules associated with the IRAK/TAB/MAPK inflammatory pathway. However, whereas 1% ECVE treatment did not upregulate the inhibitory molecules IRAK-M and pSTAT3 (Fig. 6ii, left panel), 3% ECVE treatment did upregulate these inhibitory molecules (Fig. 6ii, right panel). This may explain why IL-6 production by LPS-matured DCs is upregulated by treatment with 1% ECVE, but not with 3% ECVE.

## Discussion

The effects of E-cigarettes on the human immune system are not fully elucidated; in particular, the effects of ECVE on human DCs have not been investigated previously although numerous studies have shown deleterious (and mainly immunosuppressive) effects of CS on DCs (Alkhattabi et al. 2018; Arellano-Orden et al. 2016; Givi et al. 2016; Guinet et al. 2004; Kroening et al. 2008; Le Rouzic et al. 2016; Liao et al. 2015; Mortaz et al. 2009; Nouri-Shirazi and Guinet 2003, 2006; Nouri-Shirazi et al. 2007; Robbins et al. 2004, 2008; Stampfli and Anderson 2009; Vassallo et al. 2005). DCs are professional antigen presenting cells that link innate and adaptive immunity; therefore, due to the importance of DCs within the immune system, the aim of the present study was to investigate the effects of ECVE on the biological behaviour of DCs in vitro using human monocyte-derived DCs.

The present study showed that ECVE treatment of DCs slightly reduced the expression of some surface markers, but enhanced the production of the pro-inflammatory cytokine IL-6 by LPS-matured DCs. The ECVE treatment of immature and LPS-matured DCs also broadly upregulated their expression of signalling molecules (and other cytoplasmic molecules) associated with DC activation. Interestingly, ECVE did not upregulate IL-6 production by Poly I:C-matured DCs, nor enhance their signalling molecule expression. In fact, short-term exposure to 1% ECVE of Poly I:C-treated DCs down-regulated signalling molecule expression. It thus appears that ECVE can have different effects on DCs depending on which toll-like receptor (TLR) pathways are also stimulated (i.e. TLR-4 by LPS and TLR-3 by Poly I:C). This also relates to the type of infection that may be a co-factor, i.e. bacterial (LPS) or viral (Poly I:C).

Compared to the numerous previous reports of the effects of CS on DCs [including our own using the same experimental conditions employed here (Alkhattabi et al. 2018)], the present study indicates that ECVE has less pronounced effects than CS on DCs. This is consistent with some other studies that have compared the effects of CS and ECV on different cell types. In particular, Iskandar et al. (Iskandar et al. 2019) recently reported that, in a multi-layer toxicology system, E-liquid and E-vapour were less toxic to cultured airway epithelial cells than was CS. They found that the ‘Base’ E-liquid that is equivalent to that used in our study (containing the humectants propylene glycol and vegetable glycerin, plus nicotine), and *MESH* Classic Tobacco-flavoured E-liquid, showed similar levels of toxicity. Thus, this particular flavouring showed no additional toxicity over the Base E-liquid. However, more than 8000 different E-liquid flavourings are now available and it is probable that at least some of these will have significant respiratory and/or systemic toxicity. Indeed, some of these flavourings may be responsible for the cases of E-cigarette induced hypersensitivity pneumonitis and other lung-damaging reactions that have been reported [e.g. (Nair et al. 2019)]. Some flavourings have been shown to induce alterations in various cell types, including affecting their viability, proliferation, phagocytic ability and cytokine production (Chen et al. 2019). It would there be of interest to use flavoured E-liquids to generate ECVE and evaluate their effects on DCs.

A specific point of consistency between the effects of Base ECV on human immature and LPS-matured MoDCs in the present study, and on buccal epithelial cells in the study of Iskandar et al. (Iskandar et al. 2019) is that we observed upregulation of inflammatory signalling molecules at 30 min and 24 h and Iskandar et al. observed upregulation of an inflammatory transcriptome at 4 h and 24 h. In addition, we observed upregulation by ECVE of the release of IL-6 (but not other cytokines) from LPS-matured DCs, and they observed increased release of IL-1α and IP-10 (but not other cytokines) by buccal cells in response to Base ECV. These different studies thus provide consistent evidence for mild pro-inflammatory effects of Base ECV.

Treatment of DCs with 3% ECVE slightly, but significantly, inhibited surface expression of HLA-DR and CD86 in LPS-matured DCs. HLA-DR is an MHC II molecule that provides ‘signal 1’ in DC-T cell interaction. The co-stimulatory molecules CD80 and CD86 deliver the ‘signal 2’ to T cells: these bind to the CD28 molecule on T cells, thereby promoting their activation. Upregulation of co-stimulatory molecule levels is characteristic of mature DCs, as seen in the current study when stimulating immature DCs with LPS. Interestingly, we found that 3% ECVE attenuated CD86 expression on LPS-matured DCs, but not CD80 expression. The expression of CD80 is more abundant than CD86 on immature DCs while CD86 expression increases more rapidly and strongly than CD80 in response to stimuli that induce DC maturation (Sansom et al. 2003). Although both CD80 and CD86 can bind to CD28, CD80 in particular also interacts with CTLA-4 (Sansom et al. 2003) which inhibits T cell activation and proliferation (Krummel and Allison 1996). Compared to CD80, CD86 is a more important co-stimulatory molecule for T cell activation (Sharpe 1995) and blockade of CD86 has been reported to inhibit T cell proliferation in mixed lymphocyte reactions (Ke et al. 2016; Zheng et al. 2004), whereas blockade of CD80 promoted it (Zheng et al. 2004). In the current study, levels of CD86 but not CD80 were slightly decreased by 3% ECVE in LPS-matured DCs suggesting a potential role of this higher dose of ECVE in attenuating T cell activation by DCs.

Numerous studies have reported evidence of a role for IL-6 in inflammatory lung diseases, including hypersensitivity pneumonitis (Denis et al. 1992; Moodley et al. 2003; Park et al. 2000; Schuyler et al. 2000; Shieh et al. 2019). Several studies show that CS enhances IL-6 production by various types of cells including DCs (Chi et al. 2012; Givi et al. 2015; Wang et al. 2017), and in BALF of mice (Givi et al. 2016). In the current study, 1% ECVE significantly stimulated IL-6 secretion by LPS-matured DCs, suggesting E-cigarette use may promote an inflammatory environment in humans. However, the levels of other cytokines including IL-8, IL-10, IL-12 and TNF-α remained unchanged after ECVE treatment, although it has been reported that they are altered by CS treatment of DCs (Givi et al. 2015, 2016; Mortaz et al. 2009; Vassallo et al. 2005). It has also been reported that E-cigarette vapour condensate enhanced production of IL-6 by human alveolar macrophages (Scott et al. 2018), consistent with our findings reported here for human DCs. However, E-cigarette vapour condensate also enhanced the production of TNF-α, CXCL-8 (IL-8), MCP-1 and MMP-9 by alveolar macrophages (Scott et al. 2018). These different findings may be partly due to the different cell types being studied (i.e. alveolar macrophages versus monocyte-derived DCs) and/or the different methods of producing E-cigarette vapour extract or condensate.

Compared with other proteomic methods, RPPA is a sensitive and high-throughput technology which yields a large volume of quantitative or semi-quantitative molecular information in an experiment using only nanolitres of sample (Mueller et al. 2010). In the present study, the effects of ECVE treatment of DCs on the expression of individual signalling molecules was relatively subtle and, for most individual molecules, not statistically significant. However, as we have noted in other systems examined in this way (Negm et al. 2014), although effects on individual signalling molecules may seem relatively small, the combined or ‘global’ effects on multiple signalling molecules may be both statistically and biologically significant. Thus, looking at the global effects of ECVE, 1% ECVE generally enhanced expression of the signalling molecules at both 30 min and 24 h in immature DCs. For LPS-treated DCs, 3% ECVE significantly enhanced the expression of the signalling molecules at both 30 min and 24 h, suggesting up-regulation of signalling molecule expression as also seen in immature DCs, but with different sensitivity to ECVE concentration. In contrast, we found that 30-min treatment with 1% ECVE suppressed signalling molecule expression by Poly I:C-stimulated DCs, as we previously reported for the effects of CSE and nicotine (Alkhattabi et al. 2018).

**Fig. 7:**
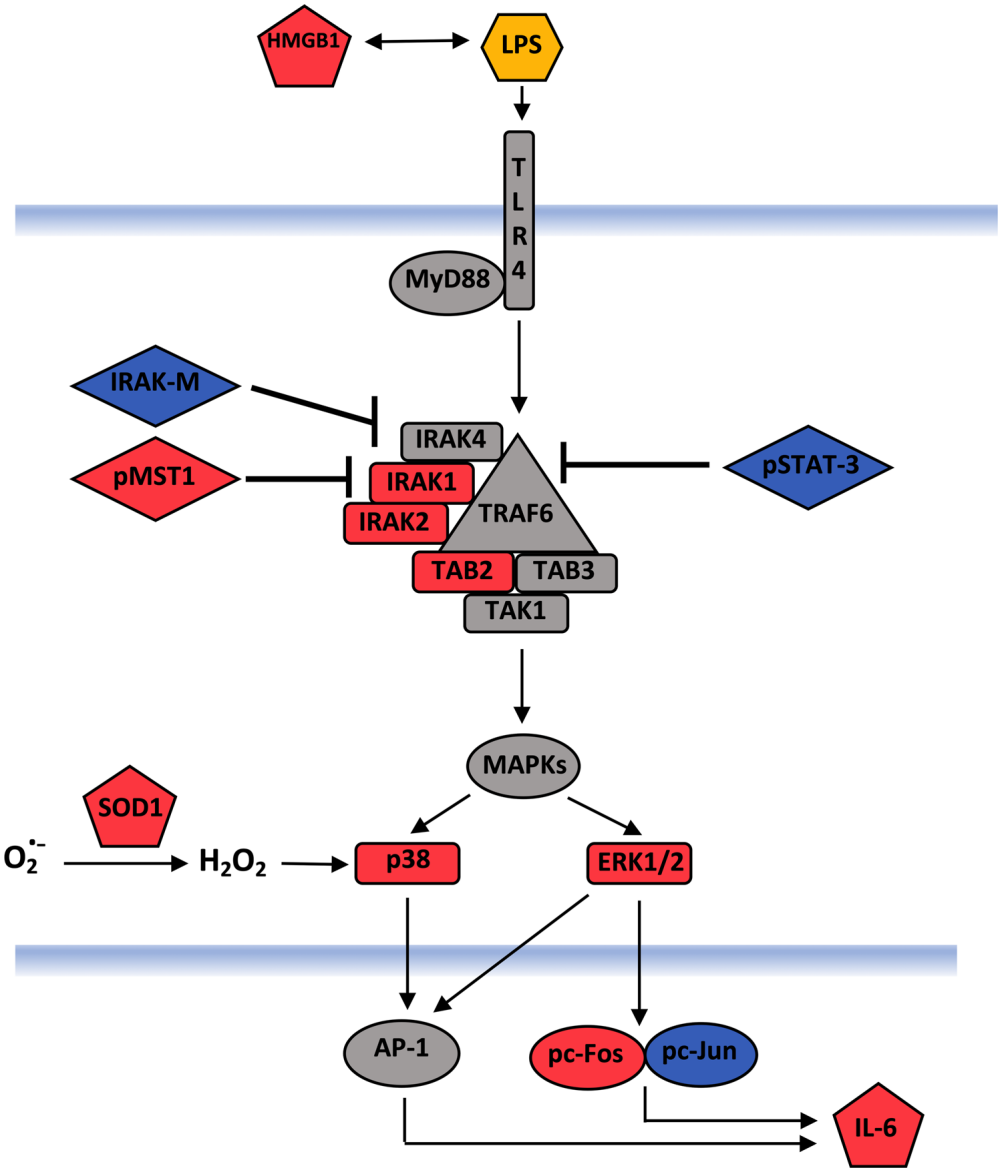
Schematic representation of the LPS-TLR4 MAPK pathway stimulated by 1% ECVE treatment of LPS-matured MoDCs. (Based on data presented in Fig. 6). Molecules that were found to be upregulated by 1% ECVE treatment are shown in red. Molecules that were unchanged or down-regulated by 1% ECVE treatment are shown in blue. Molecules that were not investigated in this study are shown in grey (color figure online)

Some signalling molecules have inhibitory/regulatory activities rather than activating/inflammatory activities in particular contexts. We thus observed that the conditions that promoted IL-6 production by DCs (i.e. 1% ECVE-treatment of LPS-matured DCs) did not give an overall statistically significant enhancement of all 29 molecules detected by RPPA at the ‘global’ level. However, these conditions did significantly upregulate molecules involved in the IRAK/TAB/MAPK inflammatory pathway, but did not upregulate certain molecules that can be inhibitory, in particular IRAK-M (Kobayashi et al. 2002) and pSTAT3 (Zhang et al. 2014) (see Fig. 7). Since the MAPK pathway is involved in maturation (Bansal et al. 2010; Dunand-Sauthier et al. 2011b; Nakahara et al. 2004; Pathak et al. 2012), cytokine production (Dunand-Sauthier et al. 2011a; Nakahara et al. 2004) and antigen-specific T cell activation (Bansal et al. 2010; Dunand-Sauthier et al. 2011a) of DCs, activation of the MAPK pathway induced by ECVE may enhance functionality of DCs. In contrast, 3% ECVE-treatment of LPS-matured DCs significantly upregulated all 29 molecules, including IRAK-M and pSTAT-3, which may explain why 3% ECVE treatment did not enhance IL-6 production. Interestingly, ECVE treatment did not consistently upregulate signalling molecules associated with the NFκB pathway (cRel and pP65), again indicating that the increased IL-6 production in response to ECVE plus LPS was probably stimulated via the MAPK pathway. We also observed that ECVE treatment upregulated DC expression of HMGB1 and SOD1: the former promotes TLR4 signalling (Bianchi 2009; Park et al. 2006) and DC maturation (Messmer et al. 2004), and the latter promotes MAPK activity via production of hydrogen peroxide (Wang et al. 2018). Overall, the global enhancement in expression of the inflammatory signalling molecules and other cytoplasmic molecules in DCs by ECVE treatment would be consistent with ECVE promoting DC functions and activity.

In conclusion, this study established an in-vitro model to investigate the effects of ECVE on human monocyte-derived DCs. Several biological aspects of DCs have been examined including signalling pathways, surface markers and cytokine production, using a variety of culture conditions. ECVE generally had moderate effects on DC function, including changed levels of signalling molecules and production of the pro-inflammatory cytokine IL-6. Therefore, E-cigarettes are not completely harmless to human DCs, but the effects appear to be less pronounced than those reported for CS.

## Electronic supplementary material

Below is the link to the electronic supplementary material.

Supplementary file1 (PDF 904 kb)
