# Supplementary material for: Electronic cigarette vapour moderately stimulates pro-inflammatory signalling pathways and interleukin-6 production by human monocyte-derived dendritic cells
Source: Arch Toxicol. 2020 May 5;94(6):2097–112. doi: 10.1007/s00204-020-02757-8 (PMC7303083; doi:10.1007/s00204-020-02757-8)
Supplement: Supplementary file 1 — Supplementary file1 (PDF 904 kb) [file 204_2020_2757_MOESM1_ESM.pdf]

SUPPLEMENTARY INFORMATION

Electronic cigarette vapour moderately stimulates pro-inflammatory signalling pathways and interleukin-6 production by human monocyte-derived dendritic cells

I-Ling Chen, Ian Todd, Patrick J. Tighe, Lucy C. Fairclough*

School of Life Sciences, University of Nottingham, Nottingham, UK

***Correspondence:**

Lucy C. Fairclough, School of Life Sciences, University of Nottingham, Life Sciences Building, University Park, Nottingham NG7 2RD, UK.

E-mail: lucy.fairclough@nottingham.ac.uk

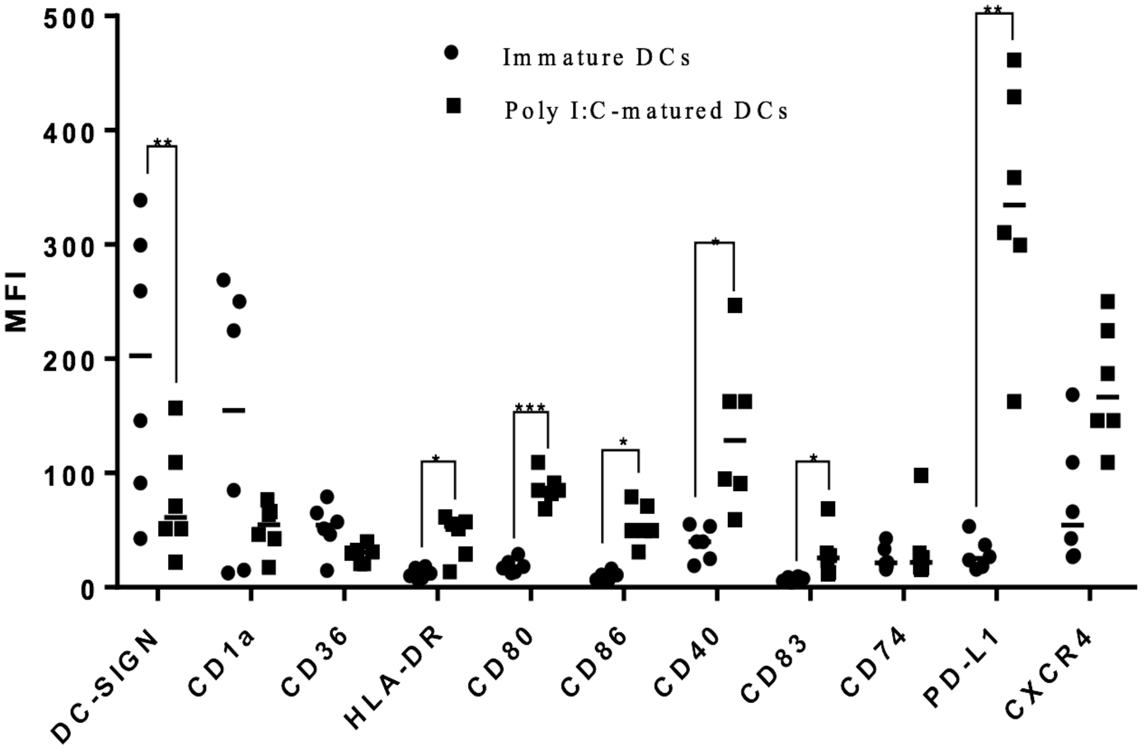

Figure 1S. Comparison of MFI value of surface markers between untreated immature and Poly I:C-matured DCs. Data is presented as scatter plots and each dot represents a different individual donor. The median of six independent experiments is shown. If data was normally distributed, paired t-test was used, otherwise Wilcoxon test was used. * p<0.05, **p<0.01, ***p<0.001, ****p<0.0001.

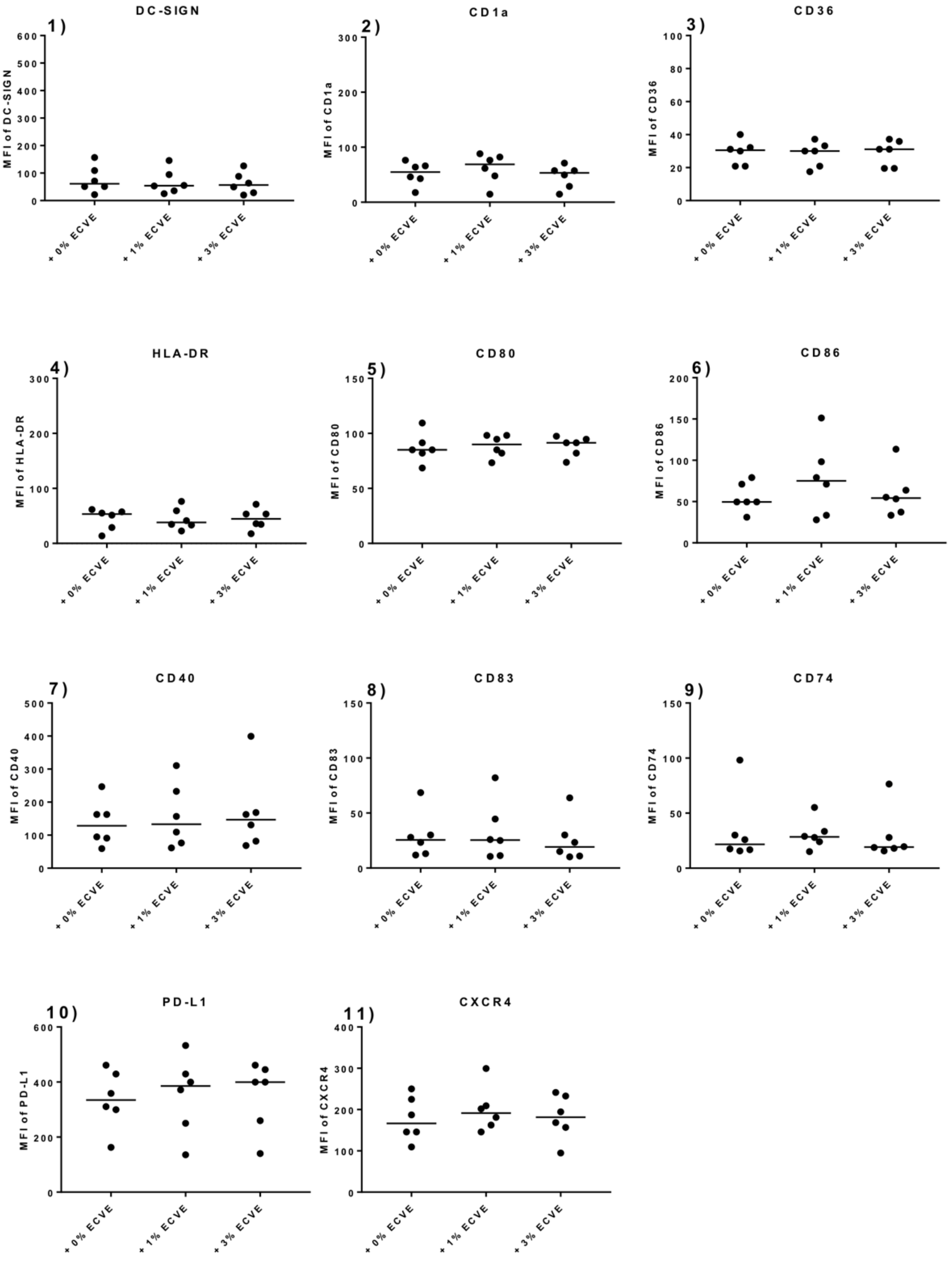

Figure 2S. The MFI of surface markers on Poly I:C-matured DCs after 24 h with or without ECVE treatment. Data is presented as scatter plots and each dot represents a different individual donor. The median of six individual experiments is shown. If data was normally distributed, paired one-way ANOVA was used, otherwise paired Friedman test was used.

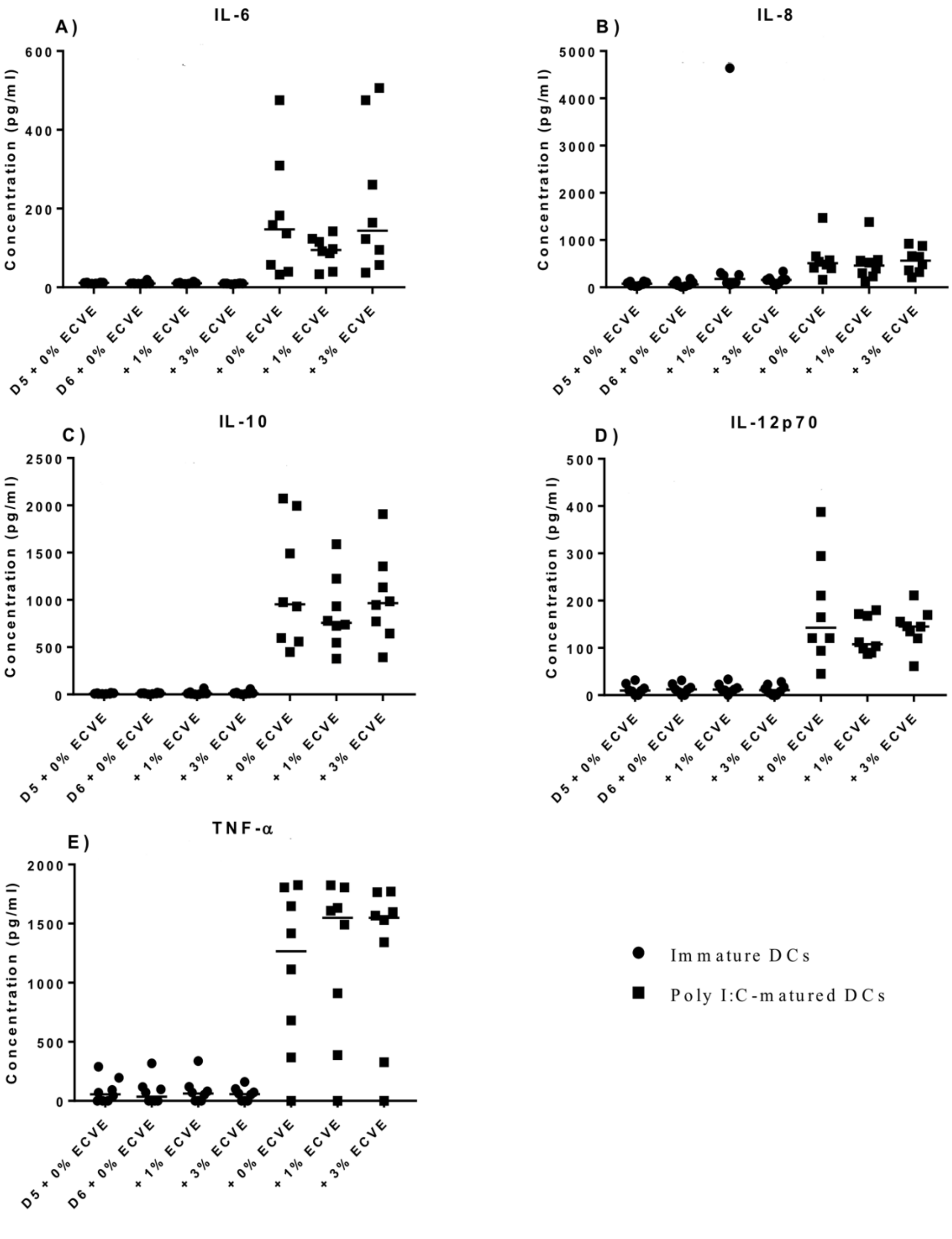

Figure 3S. Cytokine production by immature and Poly I:C-matured DCs after 24 h with or without ECVE treatment. Data is presented as scatter plots of eight independent experiments and their medians. If data was normally distributed, paired one-way ANOVA was used, otherwise paired Friedman test was used.

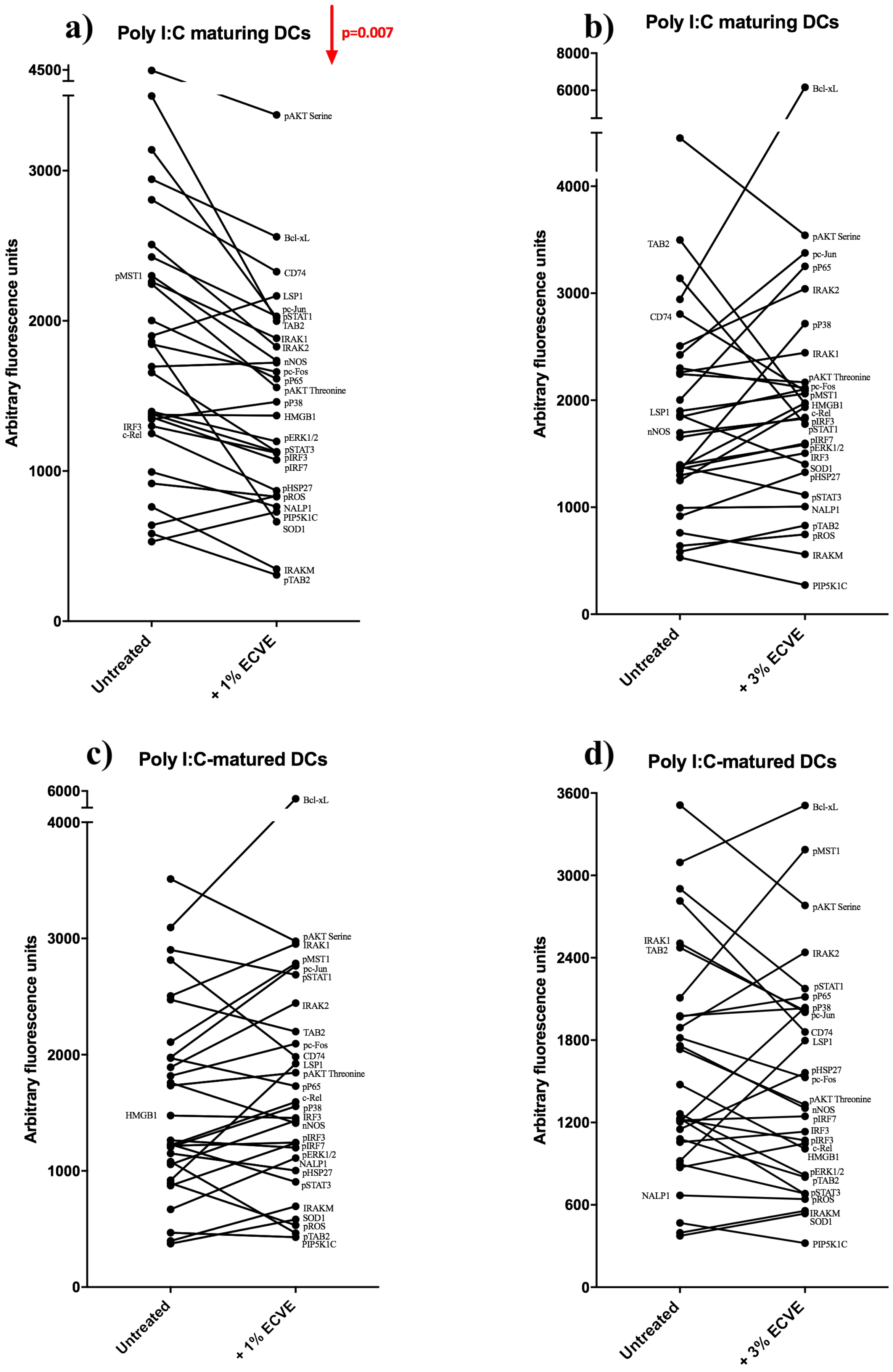
a)
Poly I:C maturing DCs
p=0.007
Arbitrary fluorescence units
4500
3000
2000
1000
0
pMST1
IRF3
c-Rel
pAKT Serine
Bcl-xL
CD74
LSP1
pc-Jun
pSTAT1
TAB2
IRAK1
IRAK2
nNOS
pc-Fos
pP65
pAKT Threonine
pP38
HMGB1
pERK1/2
pSTAT3
pIRF3
pIRF7
pHSP27
pROS
NALP1
PIP5K1C
SOD1
IRAKM
pTAB2
Untreated
+ 1% ECVE
b)
Poly I:C maturing DCs
8000
6000
4000
3000
2000
1000
0
Arbitrary fluorescence units
TAB2
CD74
LSP1
nNOS
Bcl-xL
pAKT Serine
pc-Jun
pP65
IRAK2
pP38
IRAK1
pAKT Threonine
pc-Fos
pMST1
HMGB1
c-Rel
pIRF3
pSTAT1
pIRF7
pERK1/2
IRF3
SOD1
pHSP27
pSTAT3
NALP1
pTAB2
pROS
IRAKM
PIP5K1C
Untreated
+ 3% ECVE
c)
Poly I:C-matured DCs
6000
4000
3000
2000
1000
0
Arbitrary fluorescence units
HMGB1
Bcl-xL
pAKT Serine
IRAK1
pMST1
pc-Jun
pSTAT1
IRAK2
TAB2
pc-Fos
CD74
LSP1
pAKT Threonine
pP65
c-Rel
pP38
IRF3
nNOS
pIRF3
pIRF7
pERK1/2
NALP1
pHSP27
pSTAT3
IRAKM
SOD1
pROS
pTAB2
PIP5K1C
Untreated
+ 1% ECVE
d)
Poly I:C-matured DCs
3600
3000
2400
1800
1200
600
0
Arbitrary fluorescence units
IRAK1
TAB2
NALP1
Bcl-xL
pMST1
pAKT Serine
IRAK2
pSTAT1
pP65
pP38
pc-Jun
CD74
LSP1
pHSP27
pc-Fos
pAKT Threonine
nNOS
pIRF7
IRF3
pIRF3
c-Rel
HMGB1
pERK1/2
pTAB2
pSTAT3
pROS
IRAKM
SOD1
PIP5K1C
Untreated
+ 3% ECVE

Figure 4S. The effects of ECVE-treatment on global expression of signalling molecules by poly I:C-treated DCs. Global effects on 29 signalling molecules in DCs treated with ECVE for (a, b) 30 minutes and (c, d) 24h. Data is presented as scatter plots and each dot represents the median expression of each signalling molecule from up to nine independent experiments. If data were normally distributed, one-way ANOVA was used; otherwise Freidman test was used.
